# Relationship between food waste, diet quality, and environmental sustainability

**DOI:** 10.1371/journal.pone.0195405

**Published:** 2018-04-18

**Authors:** Zach Conrad, Meredith T. Niles, Deborah A. Neher, Eric D. Roy, Nicole E. Tichenor, Lisa Jahns

**Affiliations:** 1 Grand Forks Human Nutrition Research Center, US Department of Agriculture, Agricultural Research Service, Grand Forks, North Dakota, United States of America; 2 Department of Nutrition and Food Sciences, Food Systems Program, University of Vermont, Burlington, Vermont, United States of America; 3 Department of Plant and Soil Science, University of Vermont, Burlington, Vermont, United States of America; 4 Rubenstein School of Environment and Natural Resources, University of Vermont, Burlington, Vermont, United States of America; 5 Sustainability Institute, University of New Hampshire, Durham, New Hampshire, United States of America; Massachusetts Institute of Technology, UNITED STATES

## Abstract

Improving diet quality while simultaneously reducing environmental impact is a critical focus globally. Metrics linking diet quality and sustainability have typically focused on a limited suite of indicators, and have not included food waste. To address this important research gap, we examine the relationship between food waste, diet quality, nutrient waste, and multiple measures of sustainability: use of cropland, irrigation water, pesticides, and fertilizers. Data on food intake, food waste, and application rates of agricultural amendments were collected from diverse US government sources. Diet quality was assessed using the Healthy Eating Index-2015. A biophysical simulation model was used to estimate the amount of cropland associated with wasted food. This analysis finds that US consumers wasted 422g of food per person daily, with 30 million acres of cropland used to produce this food every year. This accounts for 30% of daily calories available for consumption, one-quarter of daily food (by weight) available for consumption, and 7% of annual cropland acreage. Higher quality diets were associated with greater amounts of food waste and greater amounts of wasted irrigation water and pesticides, but less cropland waste. This is largely due to fruits and vegetables, which are health-promoting and require small amounts of cropland, but require substantial amounts of agricultural inputs. These results suggest that simultaneous efforts to improve diet quality and reduce food waste are necessary. Increasing consumers’ knowledge about how to prepare and store fruits and vegetables will be one of the practical solutions to reducing food waste.

## Introduction

Improving diet quality while simultaneously reducing environmental impact and achieving sustainable development outcomes is a critical focus globally.[1] Despite this shared international interest, progress to improve diet quality and achieve sustainable development goals related to planetary health are exceptionally challenging to achieve. The global transition toward a “Western diet”, characterized by high intake of refined carbohydrates, added sugar, sodium, and animal products, and low intake of fruits, vegetables, and whole grains, has presented simultaneous challenges for population health and environmental sustainability.[2–5] Key elements of the Western diet are among the most prominent risk factors for morbidity and mortality worldwide,[6, 7] and are major contributors to key environmental burdens such as greenhouse gas emissions and land use.[5]

Despite these challenges, achieving global sustainable development goals is critical because a growing body of research has demonstrated that healthier diets are also generally lower in their environmental impact. For example, higher quality diets have been associated with lower greenhouse gas emissions, eutrophication, water use, and cropland use.[8–11] As a result, some countries have shifted their national dietary guidelines beyond health to include indicators of sustainability. Four countries (Brazil, Germany, Qatar, and Sweden) currently include such measures in their dietary guidelines, and dozens of others have considered it,[8] including the US as recently as 2015.[12] However, the assertion that diet quality can be linked to environmental impact has been debated in recent research.[13]

Analyses linking diet quality and environmental sustainability have typically focused on a limited suite of sustainability indicators, and have not typically included food waste, despite a growing focus on understanding where and how food is wasted in the food system.[14–16] Globally, enough food is wasted every year to feed nearly 2 billion people a 2,100 kcal/day diet,[14] which amplifies the negative environmental externalities associated with agriculture and increases resource scarcity. Food waste is an important indicator of sustainability because it embodies the sum of resources used to produce uneaten food, including cropland, agricultural chemicals like fertilizers and pesticides, and irrigation water; in other words, these inputs are used to grow food that is ultimately wasted by consumers. Nitrogen fertilizer represents the single largest investment of energy in the production of many crops,[17] and circulation of reactive nitrogen can have negative effects on atmospheric conditions, in terrestrial ecosystems, in freshwater and marine systems, and on human health.[18] Phosphorus fertilizers are produced by mining finite resources of phosphate rock,[19] and can fuel harmful algal blooms when lost to the aquatic environment.[20] Pesticides have been linked to public health effects, development of pesticide resistance in pests, crop losses, bird mortality, groundwater contamination, and more.[21] Finally, irrigation practices can lead to groundwater depletion[22], water quality degradation, and competition for drinking water, among other impacts.[23] Despite this, research examining the complex relationships between diet quality, food waste, and environmental sustainability has not focused on these important measures, representing a fundamental gap in our understanding of food systems sustainability.

Here we examine the relationship between diet quality, consumer-level food waste, and multiple measures of sustainability, including use of cropland, fertilizers, pesticides, and irrigation water. The latter are predominant agricultural inputs on US farms and have strong implications for environmental burdens. Additionally, data on their application rates are publically available at the national level. We use the US as an example, given the substantial amount of food wasted: over 20% of food is lost or wasted at the consumer level each year,[24] accounting for 225–290 pounds per person per year[16, 24, 25] and 760–790 kcal per person per day.[16, 24–26] Furthermore, the US Dietary Guidelines Advisory Committee identified a need for additional investigation of the relationship between consumer behaviors, waste disposal, and the sustainability of individual food groups in order to improve long-term food security.[12] This is an important research gap that precludes a more comprehensive accounting of the multiple factors relating public health nutrition with environmental sustainability.

## Methods

Daily per capita food waste was estimated by linking various US government datasets, and these data were entered into an established biophysical simulation model to estimate the amount of cropland used to produce wasted food. Data on agricultural application rates of irrigation water, pesticides, and fertilizers were compiled from various US government datasets; and these data were combined with summary estimates of cropland waste to generate estimates of the amount of agricultural amendments used to produce uneaten food. Subsequent sections describe this approach in greater detail.

### Dietary data

Individual-level dietary data were acquired from the National Health and Nutrition Examination Survey (NHANES) waves 2007–2008, 2009–2010, 2011–2012, and 2013–2014 from 35,507 individuals 2+ y.[27] NHANES is a cross-sectional, continuous survey that collects data on demography, diet, and health behaviors from approximately 5,000 individuals per year, and data are released on a two-year cycle. NHANES is maintained by the National Center for Health Statistics. Dietary data were acquired specifically from What We Eat In America (WWEIA), the dietary component of NHANES. Individuals complete a 24-hour recall administered by a trained interviewer using United States Department of Agriculture’s (USDA) Automated Multiple Pass Method,[28] and a subset of the study population completes a subsequent 24-hour recall by telephone on a non-consecutive day. Only data from day 1 were used because this represents per capita intake (i.e., mean intake of all individuals); whereas data from multiple days of intake are needed to estimate usual intake distributions, which was not the focus here[29]. WWEIA provides dietary data as reported consumed at home and away from home by individuals, which, in most cases, is in the form of mixed dishes composed of multiple foods, such as a cheeseburger. Data on nutrient content of each mixed dish are available, but WWEIA does not disaggregate most mixed dishes into their component foods or ingredients.

### Composition of mixed dishes

The Food and Nutrient Database for Dietary Studies (FNDDS)[30] is often used to disaggregate WWEIA dishes into their component foods, but these data are not provided at the resolution needed for this study. For example, FNDDS provides information on the amount of cheese, hamburger patty, and bun in a cheeseburger, but does not provide information on the amount of individual ingredients in a bun, such as wheat flour and oil. Disaggregation of WWEIA dishes into component ingredients was achieved with the Food Commodity Intake Database (FCID),[31] which was developed by the US Environmental Protection Agency (US EPA). FCID (2005–2010) provides data on the weight of nearly 500 ingredients included in each dish listed in WWEIA. FCID is the only source of data on the amount of individual ingredients in each WWEIA dish and, therefore, represents the most comprehensive and reliable data available on dish recipes.

### Diet quality assessment

The Healthy Eating Index-2015 (HEI-2015)[32] was used to assess diet quality for each individual in WWEIA (2007–2014). HEI-2015 provides a measure of compliance with the 2015–2020 Dietary Guidelines for Americans,[33] and includes 13 components, nine of which assess adequacy (total fruit, whole fruit, total vegetables, greens and beans, whole grains, dairy, total protein foods, seafood and plant proteins, and unsaturated:saturated fats) and four of which assess moderation (refined grains, sodium, added sugars, and saturated fats). Consumption amounts of each component were acquired from the Food Patterns Equivalents Database (FPED; 2007–2014), which provides reported consumption data from WWEIA converted to HEI components.[34] All consumption amounts are standardized to a 1,000 calorie basis (except for unsaturated:saturated fats), and each component has distinct scoring standards that range from 0–5 or 0–10, with higher scores being more favorable. Moderation components are reverse scored so that ultimately greater scores are favorable for each component. The component scores were summed to compute the overall HEI-2015 score with a maximum of 100. HEI-2015 scores were computed for each individual providing dietary data in WWEIA, and individuals were grouped by quintile of HEI-2015 score, where quintile 1 represents the lowest diet quality and quintile 5 represents the highest diet quality. Mean HEI-2015 scores for each quintile were appropriately computed using the National Cancer Institute’s population-ratio method, which has been demonstrated to generate scores that more closely reflect the true population mean than the arithmetic mean of individual-level scores.[35] The population-ratio method involves calculating the ratio of the reported intake of each food group for each individual to the reported energy intake, then computing the mean of these ratios across all individuals, then computing the HEI score using this summary ratio. [35]

### Food waste data

Data on food waste were derived from the USDA Loss-adjusted Food Availability data series (LAFA).[36] LAFA is curated by USDA Economic Research Service (ERS) and provides estimates of food loss (including waste) at multiple stages in the food system for over 200 individual foods. Food loss represents the portion of food that is not consumed for any reason, including spoilage, cooking loss, and plate waste. At the consumer level, LAFA provides data on the proportion of purchased food that is inedible (such as banana peels), but does not provide information on the proportion of edible food wasted at the plate level, so these data were derived using the computations described in S1 Fig. We assumed that uneaten portions/cooking loss represents waste for the purpose of this study.

### Agricultural resource data

Cropland waste was estimated using the US Foodprint Model, a simulation model that represents the US as a closed food system.[37] The US Foodprint Model estimates cropland use associated with user-inputted data on food intake. Embedded computations use data on food losses and waste that occur at multiple points in the food system, the conversion of raw agricultural crops into edible food products, crop and grazing yields, livestock feed requirements, suitability of available land for agricultural uses, agricultural land area, and population size. Additional calculations account for multi-use crops (i.e., crops that are used to produce multiple products from equivalent mass) and multi-use cropland (cropland used to produce multiple crops during different parts of the year). Additional details are available elsewhere.[37] We modified the US Foodprint Model to produce estimates of the amount of cropland associated with food waste. Specifically, data on per capita food waste (described above) were added to the model instead of data on per capita food intake, and the embedded calculation adjusting for cooking loss and uneaten food was removed. Data on crop yields were updated to reflect the most recent five-year mean for each crop (2011–2015 for most crops),[38] and population data was updated to the 2015 US population size.[39]

National agricultural water irrigation rates, fertilizer application rates, and pesticide application rates (amount applied per land area per year) were acquired from USDA Agricultural Surveys (2002–2016)[38] and USDA Farm and Ranch Irrigation Surveys (2003–2013),[40] maintained by the National Agricultural Statistics Service (2002–2016). USDA Agricultural Surveys collect data primarily by telephone interviews with producers in all states for all major crops. Producers are selected based on the size of their operation, with larger producers having a greater likelihood of being selected to participate in the survey. Approximately 65,000–81,000 producers are surveyed for each crop annually.[41] USDA Farm and Ranch Irrigation Surveys are conducted every five years, and data are collected primarily by mailed surveys. Approximately 35,000 producers are surveyed for each data release.[40] Data for chemical use on hay and pasture are not available in USDA Agricultural Surveys, so these data were estimated based on personal communication with agricultural Extension Service agents in top producing states.

### Data compilation

**Fig 1:**
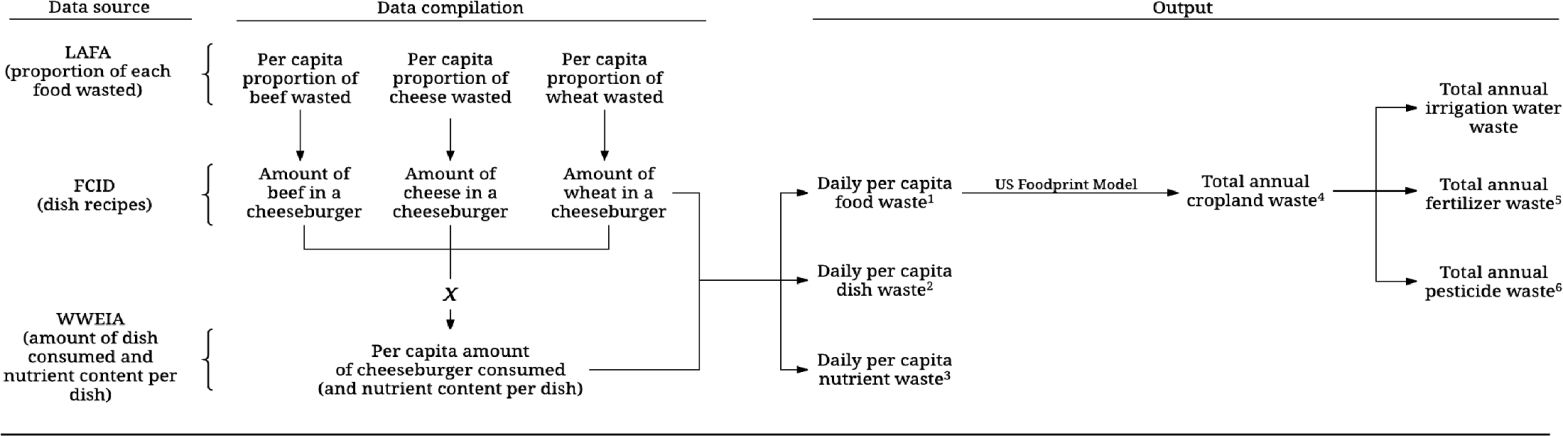
Data sources, compilation and output. LAFA, Loss-adjusted Food Availability data series; FCID, Food Commodity Intake Database; WWEIA, What We Eat In America ^1^Grains, dark green vegetables, red and orange vegetables, legumes, starchy vegetables, other vegetables. fruit, milk and yogurt, cheese and other dairy, soy milk, nuts, tofu, beef, pork, chicken, turkey, eggs, fish, plant oils, dairy fats, lard and tallow, and sweeteners. ^2^All dishes; meat and mixed meat dishes (beef and beef mixed dishes; pork and pork mixed dishes; poultry and poultry mixed dishes; seafood and seafood mixed dishes; meat sandwiches, burgers, sausages, and hotdogs; bacon; and other meat dishes) eggs and egg dishes; dairy (milk and cream, cheese); soup; grains and mixed grain dishes (bread; breakfast cereal; pancakes, waffles, and French toast; pastas and grain mixtures; pizza and calzones; and grain-based desserts); nuts and seeds; fruits and vegetables in mixed dishes (whole fruit and mixed fruit dishes; fruit/vegetable juice; dark green vegetables; yellow and orange vegetables; tomatoes and tomato mixtures; legumes; other vegetables); potatoes and potato mixed dishes; margarine, table oils, and salad dressings; salty snacks; Mexican dishes; other foods and dishes. ^3^Calories; total protein; total carbohydrates; added sugars; fiber; total and individual saturated fatty acids; total and individual monounsaturated fatty acids; total and individual saturated fatty acids; cholesterol; vitamins (A, B_1_, B_2_, B_6_, B_12_ niacin, folate, choline, C, D, K, E); minerals (calcium, phosphorous, magnesium, iron, zinc, copper, sodium, potassium, selenium); and total and individual carotenoids. ^4^All cropland, grains, fruits, vegetables, legumes, nuts, sweeteners, feed grains and oilseeds, hay, and cropland pasture. ^5^Nitrogen, phosphorus, and potash. ^6^Sum of insecticides, herbicides, and fungicides.

The proportion of each food item wasted at the consumer level (from LAFA) was linked with specific foods in FCID that best matched its description (Fig 1). Two investigators matched data independently, and consensus was used to resolve infrequent differences. Successful matches were achieved for 92% of the FCID foods; the remainder typically represented foods consumed infrequently and in minute amounts by the general population (such as passionfruit, arrowroot, and seaweed), and were not included in the analyses (S1 Table). Subsequently, linked FCID-LAFA data (which provided the proportion of each food wasted in each dish) were coupled with data on intake of mixed dishes (and their nutrient content) from WWEIA (which provided the amount of each dish and nutrient consumed) to disaggregate mixed dishes into component ingredients to estimate waste of individual ingredients, mixed dishes, and nutrients. For example, the portion size of a cheeseburger reported by a participant in WWEIA was disaggregated into its component ingredients (beef, cheese, wheat, etc.) using FCID. Each of those ingredients were then linked to a waste proportion from LAFA, which provided enough information to estimate the amount of each ingredient wasted for each individual in our WWEIA sample (S1 Fig).

The wasted amount (by weight) of individual ingredients were grouped according to the categorization scheme used in the US Foodprint model, which includes 22 distinct food categories. Mixed dishes were grouped primarily according to the FNDDS categorization scheme, which resulted in 36 distinct mixed dish categories. In cases where a mixed dish included disparate foods (e.g., meat and grains), the mixed dish was categorized in FNDDS according to the predominant food in the mixed dish (for example, steak with a side of rice was categorized as a mixed meat dish). The amount of nutrients associated with food waste was estimated for 57 individual nutrients.

For each individual in our WWEIA sample, HEI-2015 scores were computed, and the entire sample was divided into quintiles based on HEI-2015 scores. The mean waste of each ingredient was estimated for each HEI-2015 quintile, and these data were entered into the US Foodprint Model to estimate the annual amount of cropland associated with food waste, by HEI-2015 quintile, which was reported for ten distinct cropland uses. Estimates of cropland amounts associated with food waste were applied to application rates of irrigation water, fertilizers (nitrogen, phosphorus-P_2_O_5_, and potash-K_2_O), and pesticides for major cropland uses to estimate waste of agricultural resources.

### Analysis

The relationship between daily per capita food waste amount (grams) and HEI-2015 quintiles was estimated using simple linear regression models to test for trend, with food waste as the dependent variable and quintile of diet quality as the independent variable. Statistical significance was set at *P*<0.05. All analyses were adjusted for the complex, multistage, probability sampling design of WWEIA using standardized procedures and variables provided by the National Center for Health Statistics.[42] Total annual amount of cropland associated with food waste was estimated using the US Foodprint model. A Monte Carlo simulation with 1,000 random, non-replacement draws was embedded into the US Foodprint model to provide measures of variation, based on inter-individual variation in reported food intake from WWEIA. The relationship between each of the sustainability measures (cropland use, and application of irrigation water, pesticides, and fertilizers) and HEI-2015 quintiles was estimated using simple linear regression models to test for trend, with a given sustainability measure as the dependent variable and quintile of diet quality as the independent variable. Statistical significance was set at *P*<0.05. SAS 9.4 (SAS Institute; Cary, NC) was used to estimate population-ratio HEI-2015 scores using the modified code and macros provided by the National Cancer Institute.[43] Stata14 (StataCorp; College Station, TX) was used for data management and all other analyses.

## Results

### Daily per capita food and nutrient waste

US consumers wasted 422 g (95% CI: 409–434 g)–nearly one pound–of food per person per day from 2007–2014 (Table 1 and S2 Table). Fruits and vegetables and mixed fruit and vegetable dishes accounted for 39% of food waste, followed by dairy (17%), meat and mixed meat dishes (14%), and grains and grain mixed dishes (12%). Remaining foods and dishes each accounted for less than 10% of total food waste: other foods and dishes (mostly candy, soft drinks, and other beverages), salty snacks, soup, potatoes and mixed potato dishes, nuts and seeds, Mexican dishes, eggs and mixed egg dishes, and table oils and salad dressing.

**Table 1:** Daily per capita food waste (n = 35,507).

Food or dish item	Mean (95% CI), grams		Percent
Total	421.5	(409.1–433.9)	100.0
Fruits and vegetables and mixed fruit and vegetable dishes	163.9	(153.9–173.8)	38.9
Dairy	72.3	(70.2–74.3)	17.1
Meat and mixed meat dishes	56.8	(55.0–58.6)	13.5
Grains and mixed grain dishes	50.8	(44.5–57.2)	12.1
Other foods and dishes^1^	24.5	(23.7–25.3)	5.8
Salty snacks	15.6	(14.5–16.8)	3.7
Soup	11.8	(10.5–13.2)	2.8
Potatoes and mixed potato dishes	8.6	(8.3–9.0)	2.0
Nuts and seeds	5.8	(2.4–9.2)	1.4
Mexican dishes	5.4	(4.9–6.0)	1.3
Eggs and mixed egg dishes	2.8	(2.6–3.0)	0.7
Table oils and salad dressing	2.2	(2.1–2.3)	0.5

^1^Mostly candy, soft drinks, and other beverages.

Nearly 26% (95% CI: 25–26%) of food was wasted by US consumers every day from 2007–2014 (S3 Table). Soup, fruits and vegetables and mixed dishes, and other foods and dishes had the highest waste rate (approximately 30% each). Nuts and seeds, potatoes and mixed potato dishes, and table oils and salad dressing had the lowest rates of food waste (12–18% each). Over 800 kcal (795–840 kcal) were wasted per person per day, representing about 29% of total daily energy intake (S4 Table). Of all nutrients, carotenoids had the greatest percent waste (31%) and vitamin D had the lowest percent waste (25%).

### Annual cropland and agricultural amendments used to produce wasted food

**Fig 2:**
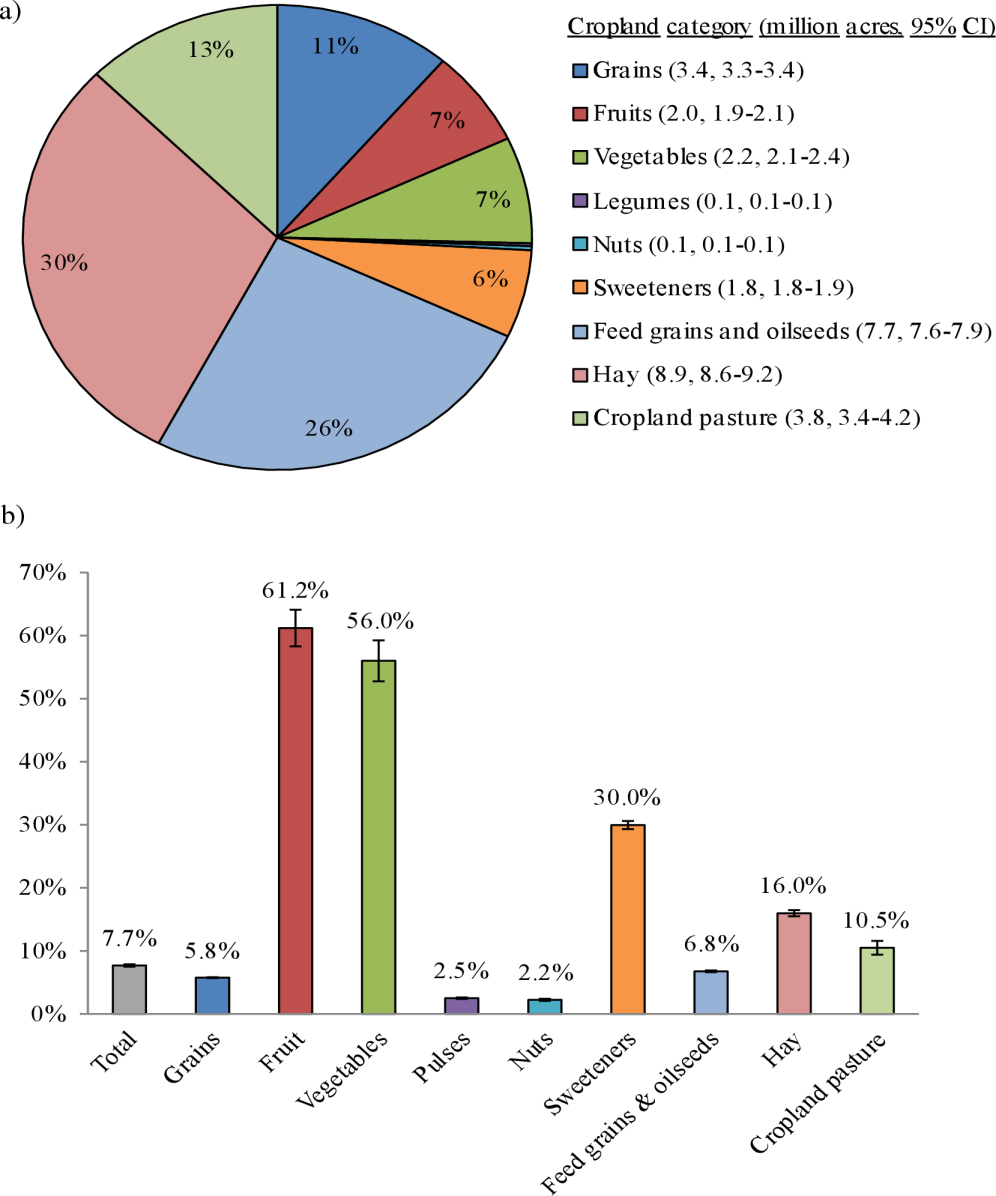
a) Percent of all harvested cropland wasted by category, and b) percent of each type of harvested cropland wasted. Total cropland wasted = 30.02 million acres (95% CI: 29.29–30.76 million acres), representing 7.7% (7.5–7.9%) of total harvested cropland.

Annually, wasted food was grown on the equivalent of over 30 million acres (95% CI: 29.3–30.8 million acres) of cropland, representing 7.7% (7.5–7.9%) of all harvested cropland in the US (Fig 2A). Hay (8.9 million acres, 6.9–9.2 million acres) and feed grains and oilseeds (7.7 million acres, 7.6–7.9 million acres) accounted for over half (56%) of all cropland used to produce wasted food. Over 60% (58–64%) of land used to grow fruit was wasted, followed by vegetables (56%, 52–59%), and sweeteners (30%, 29–31%; Fig 2B). Cropland categories with the lowest proportion of waste were nuts (2.3%, 2.1–2.4%) and legumes (2.5%, 2.4–2.6%).

Nearly 4.2 trillion gallons (95% CI: 4.1–4.3 trillion gallons) of irrigation water were applied to cropland that was used to produce uneaten food (S5 Table). The majority of wasted irrigation water was applied to cropland used to produce fruits (1.3 trillion gallons), vegetables (1.05 trillion gallons), and hay (1.01 trillion gallons). Nearly 780 million pounds (759–797 million pounds) of pesticides were applied to wasted cropland (S6 Table), mostly to cropland used to produce fruit (337 million pounds), feed grains and oilseeds (158 million pounds), and vegetables (133 million pounds). Approximately 1.8 billion pounds (1.8–1.9 billion pounds) of nitrogen fertilizer (S7 Table), 1.5 billion pounds (1.4–1.5 billion pounds) of phosphorus (P_2_O_5_) fertilizer (S8 Table), and 2.3 billion pounds (2.2–2.3 billion pounds) of potash (K_2_O) fertilizer (S9 Table) were applied to wasted cropland, largely attributable to cropland used to produce feed grains and oilseeds and hay.

### Daily per capita food waste and diet quality

**Fig 3:**
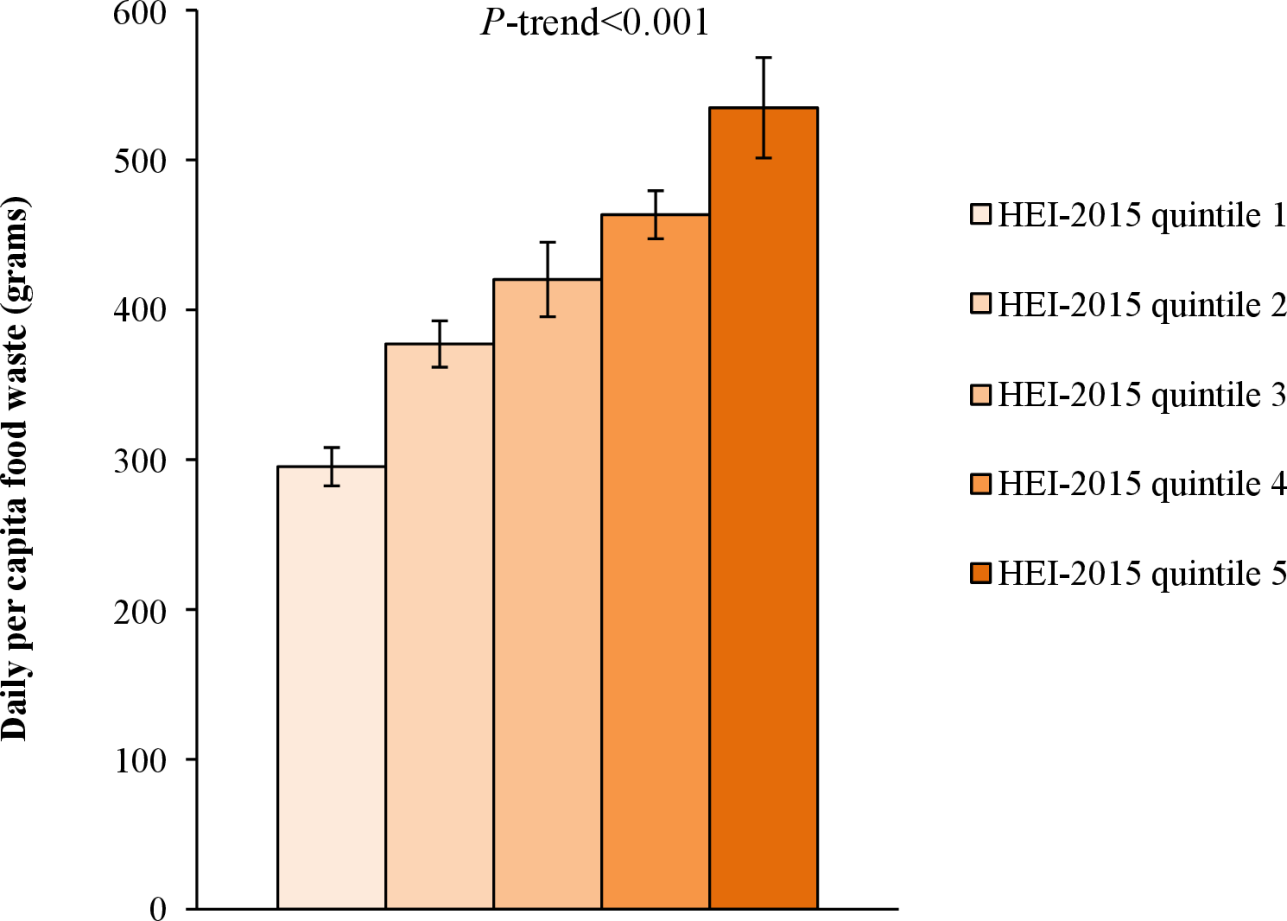
Total food waste by Healthy Eating Index-2015 quintile. HEI-2015, Healthy Eating Index-2015. Higher HEI-2015 quintiles indicate higher diet quality.

The mean overall HEI-2015 score measuring diet quality was 58 (out of 100), and ranged from a mean of 32 in quintile 1 to a mean of 82 in quintile 5 (S10 Table). Higher diet quality was associated (*P*<0.001) with greater food waste (Fig 3). Food waste varied from a mean of 295 g (95% CI: 282–308 g) among those with the lowest diet quality (quintile 1) to a mean of 535 g (501–568 g) among those with the highest diet quality (quintile 5). Among individual foods and dishes, higher diet quality was associated with greater waste of dairy (*P*<0.001), soup (*P* = 0.001), nuts and seeds (*P* = 0.017), fruits and vegetables and mixed dishes (*P*<0.001), table oils and salad dressing (*P*<0.001), and salty snacks (*P*<0.001; S1 Table).

**Fig 4:**
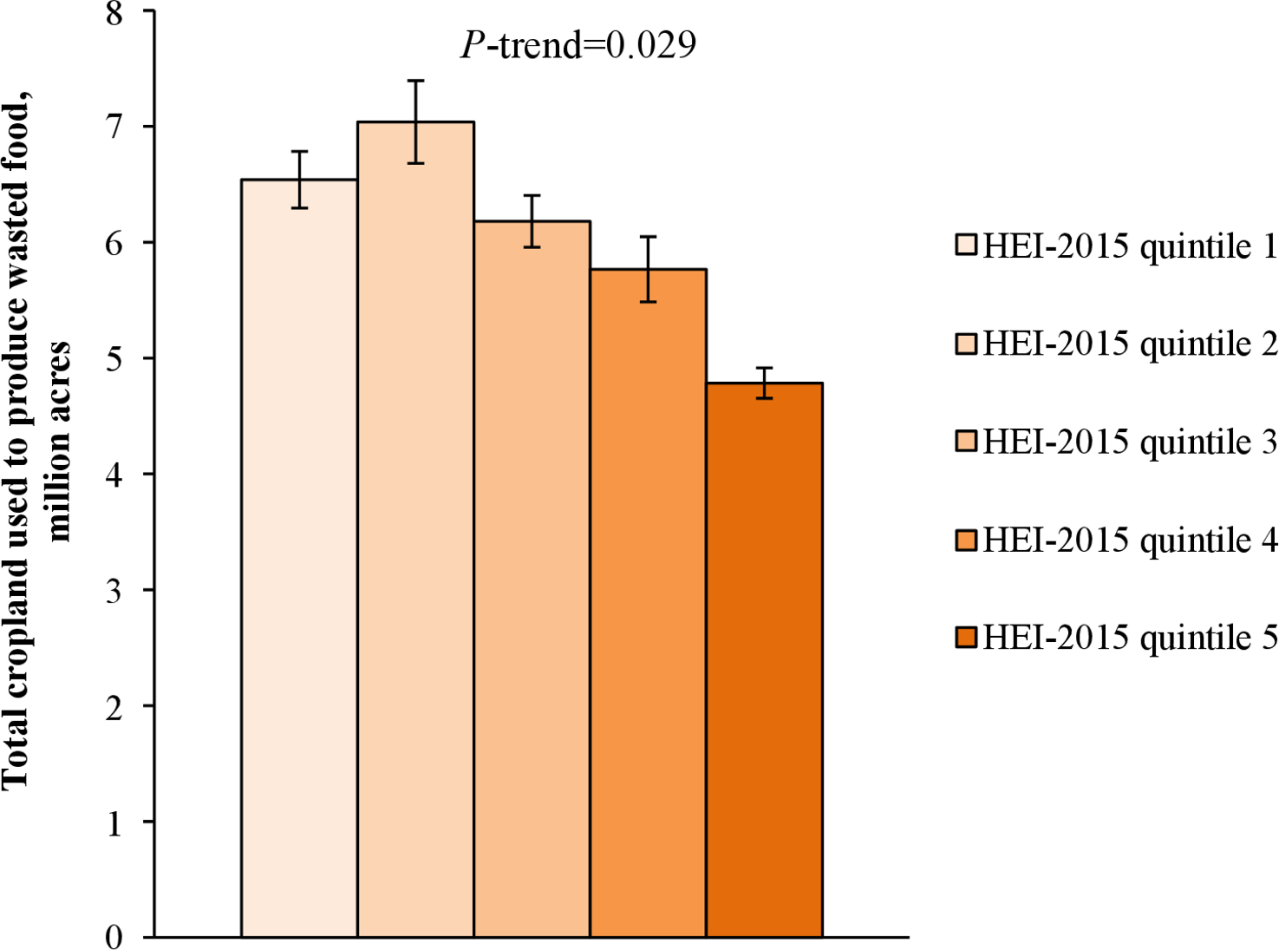
Total cropland used to produce wasted food, by diet quality. HEI-2015, Healthy Eating Index-2015.

**Fig 5:**
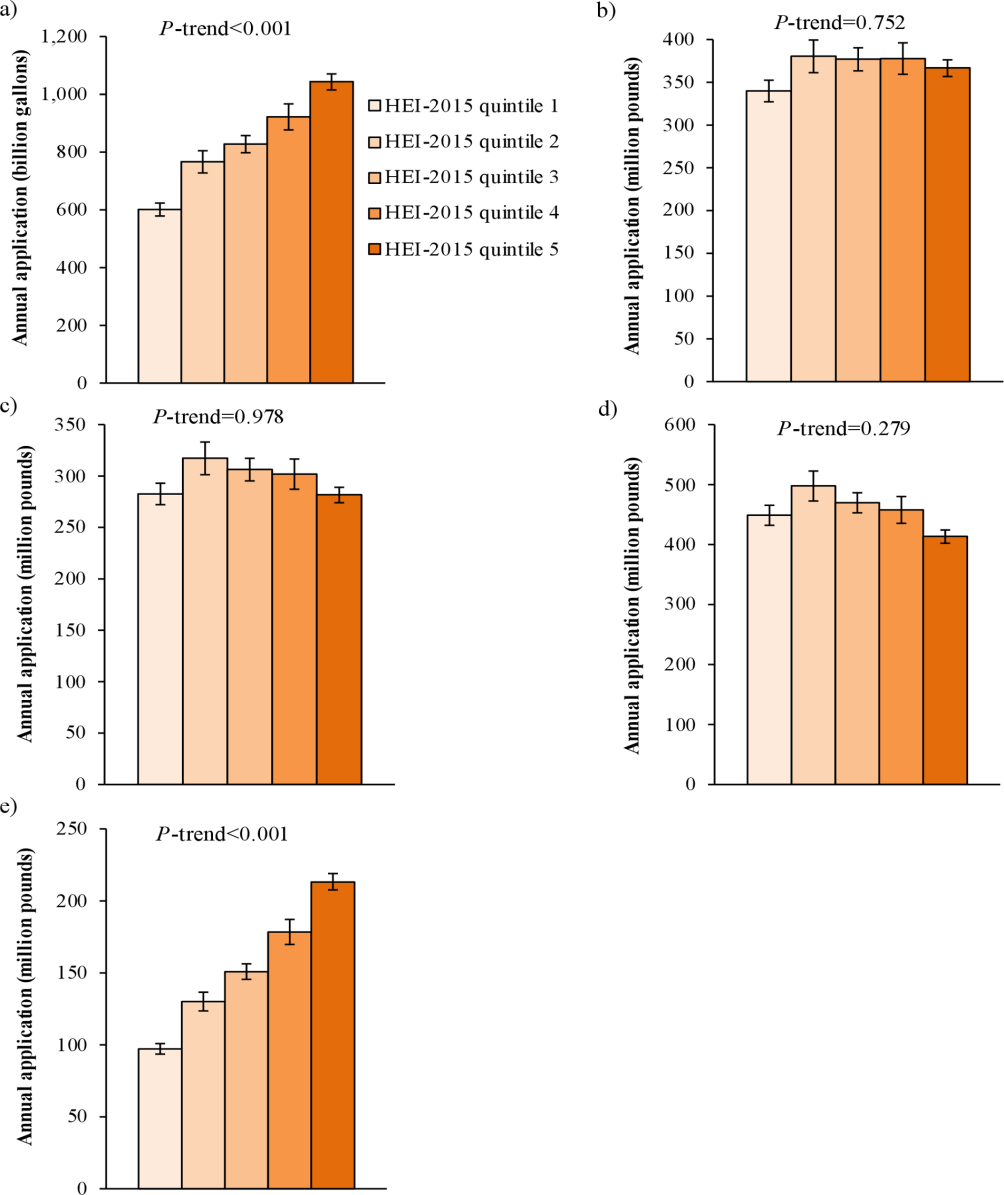
Annual amount of a) irrigation water, b) nitrogen fertilizer, c) phosphorus (P_2_O_5_) fertilizer, d) potash (K_2_O) fertilizer, and e) pesticides applied to cropland used to produce wasted food, by diet quality.

Higher diet quality was associated (*P* = 0.029) with less cropland used to produce wasted food (Fig 4). In particular, higher diet quality was associated with less land used to produce wasted grains (*P* = 0.004), sweeteners (*P*<0.001), and hay (*P* = 0.017), and more land used to produce wasted fruits (*P* = 0.001), vegetables (*P* = 0.010), legumes (*P* = 0.032), and nuts (*P* = 0.042; S11 Table). Higher diet quality was also associated with greater waste of irrigation water (*P*<0.001) and pesticides (*P*<0.001; Fig 5).

## Discussion

This is the first study, to the best of our knowledge, to integrate diverse analytical methods from the fields of nutritional epidemiology, agricultural science, and biophysical modeling to investigate the relationship between consumer food waste, diet quality, nutrient waste, and embodied agricultural resources. These results are robust and novel given this interdisciplinary context, the large size of our dataset, and the timely nature of the topic and data sources. We demonstrate that the average American wasted about 422 g (~ 1 pound) of food every day from 2007–2014, and about 30 million acres of cropland were used to produce this food every year. This accounts for about 30% of daily calories available for consumption, one-quarter of daily food (by weight) available for consumption, and 7% of annual cropland acreage. Importantly, higher quality diets were associated with greater food waste but less waste of cropland; as well as greater waste of irrigation water and pesticides, but not fertilizers.

The conventional wisdom has been that higher quality diets have less environmental impact,[10] although others cast doubt on this assertion.[13] Our findings add critical nuance to this debate. Until now, there has been limited research on the relationship between diet quality and food waste, representing a critical research gap in the field of food system sustainability. Past investigators have highlighted the land, irrigation water, greenhouse gas, and reactive nitrogen burdens of producing animal-sourced foods, especially beef.[44, 45] However, as our results highlight, production of fruits and vegetables wasted in high proportions carries environmental burdens as well, particularly due to relatively high rates of pesticide use and irrigation. Higher quality diets contained greater amounts of fruits and vegetables, which require far less land to produce compared to many other foods.[46, 47] Yet a substantially greater proportion of fruits and vegetables were wasted compared to other foods, and fruits and vegetables have higher agricultural input needs (per unit of land area) than most other crops.[38] Fruits and vegetables are also more likely to be purchased raw than other foods, transferring more of the processing activity and waste to the consumer phase of the food system rather than to the processor (pre-consumer) phase. Thus, diet quality and environmental sustainability are not necessarily interdependent, and improving diet quality and reducing environmental impact are efforts that should be pursued concurrently: consumers should increase their consumption of fruits and vegetables and simultaneously waste less of them.

In this study we used a novel methodology for estimating the amount of daily food waste that makes our findings distinct from earlier research. Up until now, the amount of food lost and wasted in the US has been estimated using the USDA Loss-adjusted Food Availability data series (LAFA) as the underlying data on food intake. For example, Buzby et al.[24] estimated 360 g/d per capita daily food loss and waste (compared to 422 g/d in the present study) and Spiker et al.[26] estimated 759 kcal per capita daily loss and waste (compared to 817 kcal in the present study). In the present study, we used data on food intake from WWEIA, the only nationally-representative data on self-reported food intake in the US. This is important because WWEIA includes more foods than LAFA and, therefore, likely better represents total dietary intake; and also provides data on foods as they were reported consumed (i.e., mixed dishes rather than individual ingredients), thereby making our estimates relevant to actual food consumption patterns. While self-reported dietary intake data are subject to measurement error (for example, some participants may not accurately report their food intake in order to simplify the survey process or to impress the interviewer) [48, 49], they are particularly useful for comparing dietary patterns between groups.[50]

Our estimates of cropland (30 million acres) and fertilizer (5.6 billion pounds) used to produce wasted food were lower than Kummu et al. (44 million acres of cropland and 7.3 billion pounds of fertilizer),[14] even though they did not include animal-based foods in their analysis (i.e. meat, dairy, eggs, hay, and pasture), which are primary drivers of cropland use and agricultural inputs.[37, 46] This divergence can likely be explained, in large part, by differences in scope: Kummu et al. did not focus exclusively on the US (data on North America and New Zealand were aggregated) and did not restrict their findings to consumer level waste (all post-harvest losses were aggregated). Our estimates of cropland and fertilizer used to produce wasted food were also lower than Birney et al. (61 million acres of cropland and 8.7 billion pounds of fertilizer),[51] even though they, too, did not account for the land use associated with production of dairy, oilseed, and livestock feed, and did not account for the amount of fertilizers associated with livestock feed production. In contrast to our analysis, Birney et al. included retail food loss and waste in their analysis and did not account for multi-use crops (i.e., crops that are used to produce multiple products from equivalent mass) and multi-use cropland (cropland used to produce multiple crops during different parts of the year), both of which could explain the observed differences in results.

We also show that substantial amounts of nutrients are embodied in wasted food. It is tempting to assume that these wasted nutrients would have been consumed (had they not been thrown away) in addition to the food that was actually consumed, representing a nutritional boon for consumers and an opportunity to improve diet quality.[26] Yet we recommend against this interpretation because this would also lead to higher intake of overconsumed nutrients like saturated fat, sodium, and added sugar, which have been linked to negative health outcomes.[52] And given limited time, perishability, and other logistical challenges, it is not feasible to prevent all food waste at the consumer level.[53] More to the point, for many Americans with adequate financial resources it is likely that, when food was discarded, other food was purchased and consumed in its place in order to prevent hunger. By contrast, for individuals with limited food budgets, wasted food may not be replaced with other foods and may indeed contribute to hunger. Some have suggested that purchasing less perishable foods may lead to less food waste,[54] like canned and packaged foods; but in so doing individuals should also choose foods low in saturated fat, sodium, and added sugar.

Consumers face a delicate balance between following dietary recommendations to increase their consumption of fruits and vegetables (which requires purchasing more of them) while also wasting less of them. This can be especially challenging for individuals with limited time and money, including families with children who face competing food preferences in the household.[54] Increased efforts to plan food purchases based on household food stocks is one way that consumers can reduce food waste due to over-purchasing;[53, 54] and increasing consumers’ knowledge about how to tell when fruits and vegetables are ripe, how to store and prepare them, and how to tell the difference between bruises/abrasions and spoilage will be critically important to reducing food waste.[15, 24] In a recent study, researchers found a positive relationship between the amount of food selected at each meal and the amount of plate waste, suggesting a need to better align satiety levels with food portioning.[55] Additionally, consumers can preserve some types of fruits and vegetables by freezing or canning, and should be advised that consuming frozen and canned fruits and vegetables can be a healthy way to meet dietary recommendations. This information could be emphasized in key consumer outreach programs like the Supplemental Nutrition Assistance Education Program, which provides education and guidance for low-income households to make healthy food choices.

Our findings also highlight the need for greater efforts to reduce household waste of packaged but perishable goods like dairy. For these products, substantial waste may be generated by consumers’ limited understanding of date labels on food packages (e.g. “sell by”, “use by”, and “best before”) that leads them to discard unspoiled food.[24, 56] Several novel technologies have recently been developed to help consumers identify whether foods are safe to eat, such as sensors on food packages that detect spoilage;[57] and apps have been developed to remind consumers of date labels and help plan meals to avoid food waste.[58, 59] Legislative efforts, such as the proposed Food Date Labeling Act of 2016, aim to standardize date labels on foods to reduce consumer confusion and food waste,[60] yet the fate of this proposed legislation is uncertain. At the same time, voluntary industry standards for date labels have been adopted by some of the largest US trade groups for the grocery industry.[61] It is also important to ensure that efforts to reduce food waste at the consumer level do not undermine legitimate food safety concerns. Spoiled food is a health risk, particularly for young children with under-developed immune systems, as well as for people over age 65 who may experience age-related declines in sensory function and must rely on date labels. [62, 63]

In some circumstances, consistent clinical recommendations to increase diet quality may lead individuals to purchase more healthful goods, like fruits and vegetables, which may go ultimately uneaten without tandem efforts to support healthy diets and reduce waste at the institutional, programmatic, and legislative levels, like those mentioned above. But practically, not all consumer waste can be prevented. Most households face competing challenges between the cost of food, the time and energy need to prepare and store food, diverse taste preferences among members of the household (particularly children), and other practical considerations.[24] Realistically, it may not be possible to prioritize food waste avoidance in households with other competing priorities.

This study includes several limitations. Due to lack of data availability, our estimates of food waste included cooking losses, such as fats and oils left in the pan after cooking. While some may consider this uneaten food to be wasted, others may not, so these results should therefore be interpreted as the best available evidence rather than perfect estimates. While we recognize that food waste rates likely vary in ways not captured by LAFA, this dataset is the most comprehensive and contemporary data available at the national level. The modeling approach we used assumed that all food used to feed US consumers was domestically grown, which does not reflect the globalized nature of the current food system. We used this approach because the US provides high quality, publically available data on agricultural inputs for individual crops rather than for only aggregated crop categories. We believe this approach led to greater accuracy in computing point estimates and variances than would have been possible had we used more aggregated data from international sources. The US agriculture sector is also among the most productive in the world,[64] so our approach may have underestimated the amount of cropland used to produce wasted food. Finally, self-reported dietary data are subject to measurement error, such that some individuals may over-report consumption of perceived healthy foods and under-report consumption of perceived unhealthy foods.[48, 49] This may have led to an over-estimation of total food waste (given that fruits and vegetables, which are widely understood to be health-promoting, are wasted at relatively high rates), but self-reported dietary data are still useful for comparing dietary patterns between groups.[50]

This work provides a first look at the connection between diet quality and food waste, and further research is needed to improve our understanding of these complex relationships. Building on this approach, further research should include additional indicators of sustainability (e.g., soil erosion and biodiversity) to better explore the tradeoffs between meeting human food needs and environmental impacts. Additional research is also needed to critically compare the effectiveness of specific interventions to reduce and recover food waste at multiple stages of the food system; and to better understand how wasted food can be recycled into usable goods, such as agricultural compost, or diverted to anaerobic digesters for biogas production, and the environmental benefits associated with these methods. From a social science perspective, research is needed to better understand the monetary cost of food waste at the household level, which is particularly important for households with limited food budgets.

## Conclusion

Using novel, interdisciplinary methods, we demonstrate that the average person living in America wastes nearly one pound of food daily. Furthermore, we find that higher diet quality is associated with greater food waste but less cropland waste, and greater waste of agricultural irrigation water and pesticides. This is largely due to the greater amount of fruits and vegetables included in higher quality diets, which have higher rates of waste, lower cropland needs, and higher application rates of agricultural inputs compared to other crops. Food waste is a critical component of environmental sustainability that, until now, has not been rigorously analyzed alongside diet quality. The current results suggest that simultaneous efforts to improve diet quality and reduce food waste may be critical. Practically, increasing consumers’ knowledge about how to prepare and store fruits and vegetables will be an essential component to reducing food waste. A number of important efforts have been proposed or are underway to reduce and repurpose food waste at the individual and institutional levels, yet further research is needed to better understand the comparative effectiveness of these efforts. Additional research is also needed to better understand how reducing food waste can contribute to monetary savings at the household level, especially for those with limited food budgets.

## Supporting information

S1 Fig Steps to derive the proportion of food waste from the edible weight of food. Text boxes with solid outlines represent data acquired from USDA Loss-adjusted Food Availability data series (LAFA); text boxes with dashed outlines represent derived data.
(TIFF)

S1 Table Dataset linkage for the Loss-adjusted Food Availability data series and Food Commodity Intake Database. (XLSX)

S2 Table Daily per capita food waste, overall and by Healthy Eating Index-2015 quintile (n = 35,507). (XLSX)

S3 Table Proportion of food and dish items wasted (n = 35,507). (XLSX)

S4 Table Nutrients embodied in food waste, per person per day (n = 35,507). (XLSX)

S5 Table Annual amount of irrigation water used to produce wasted food, overall and by Healthy Eating Index-2015 quintile (n = 35,507). (XLSX)

S6 Table Annual amount of pesticides used to produce wasted food, overall and by Healthy Eating Index-2015 quintile (n = 35,507). (XLSX)

S7 Table Annual amount of nitrogen fertilizer used to produce wasted food, overall and by Healthy Eating Index-2015 quintile (n = 35,507). (XLSX)

S8 Table Annual amount of phosphorus (P_2_O_5_) fertilizer used to produce wasted food, overall and by Healthy Eating Index-2015 quintile (n = 35,507). (XLSX)

S9 Table Annual amount of potash (K_2_O) fertilizer used to produce wasted food, overall and by Healthy Eating Index-2015 quintile (n = 35,507). (XLSX)

S10 Table Healthy Eating Index-2015 component scores, national health and nutrition Examination Survey, 2007–2014 (n = 35,507). (XLSX)

S11 Table Annual cropland used to produce wasted food, overall and by Healthy Eating Index-2015 quintile (n = 35,507). (XLSX)
