# Supplementary figures and images for: Relationship between food waste, diet quality, and environmental sustainability
Source: PLoS One. 2018 Apr 18;13(4):e0195405. doi: 10.1371/journal.pone.0195405 (PMC5905889; doi:10.1371/journal.pone.0195405)

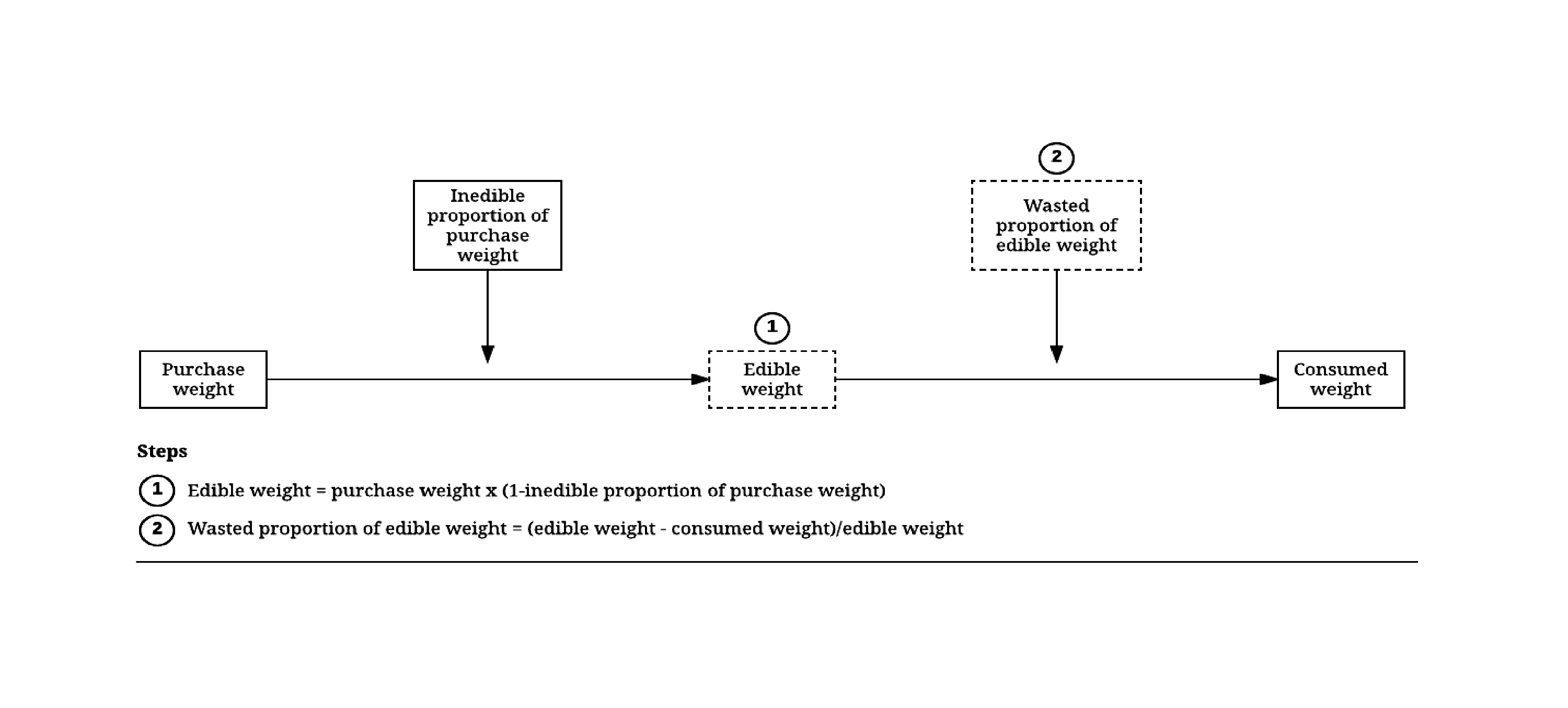

Supplement: S1 Fig — Text boxes with solid outlines represent data acquired from USDA Loss-adjusted Food Availability data series (LAFA); text boxes with dashed outlines represent derived data. (TIFF) [file pone.0195405.s001.tiff]
